# Deletion of RNF186 expression suppresses diet-induced hepatic steatosis by regulating insulin activity

**DOI:** 10.1016/j.isci.2022.103859

**Published:** 2022-02-02

**Authors:** Xiuqi Hu, Qifan Zhang, Manyu Guo, Qianqian Yuan, Xin Tong, Qing Zhang, Li Lin, Lei Zhang, Shujuan Lv, Xiaojun Liu, Chaobing Gao, Yongsheng Chang, Huabing Zhang

**Affiliations:** 1 Department of Biochemistry and Molecular Biology, Metabolic Disease Research Center, School of Basic Medicine, Anhui Medical University, Hefei 230032, China; 2 Department of Physiology and Pathophysiology, Tianjin Medical University, Tianjin 300070, China; 3 Department of Otorhinolaryngology Head and Neck, First Affiliated Hospital of Anhui Medical University, Hefei 230022, China; 4 Department of Microbiology, School of Basic Medical Sciences, Anhui Medical University, Hefei 230032, China; 5 National Laboratory of Medical Molecular Biology, Institute of Basic Medical Science, Chinese Academy of Medical Science and Peking Union Medical College, Beijing 100005, China

**Keywords:** NAFLD, RNF186, Lipid metabolism, Insulin resistance, Inflammation

## Abstract

RING finger protein186 (RNF186) is dramatically upregulated in steatotic livers. The physiological role of RNF186 in non-alcoholic fatty liver disease (NAFLD) remains obscure. Here, we found that hepatocyte-specific RNF186 knockout (RNF186^*LKO*^) mice were protected from HFD-induced obesity. RNF186 ablation in liver suppressed inflammatory responses and ER stress and alleviated insulin tolerance, leading to improved glucose and lipid metabolism under HFD conditions. RNA-seq and western blot analyses revealed a significant downregulation of peroxisome proliferator-activated receptor γ, stearoyl-CoA desaturase 1, and cluster of differentiation 36 in the liver of RNF186 knockout mice consuming HFD. RNF186 deletion in liver results in less weight gain during HFD feeding and is associated with reduced liver fat, inflammation, and improved glucose and insulin tolerance. In contrast, upregulation of RNF186 in C57BL/6J mice livers impaired lipid metabolism and insulin tolerance. The collective results suggest that RNF186 may be a potential regulator of NAFLD in obesity.

## Highlights

• RNF186 deficiency on high-fat diet alleviates liver steatosis and insulin tolerance
• RNF186 increased hepatic TG accumulation and impaired insulin sensitivity
• RNF186 ablation suppresses hepatic inflammation associated with high-fat diet
• RNF186 maybe a potential regulator of NAFLD in obesity

## Graphical abstract

**Figure:**
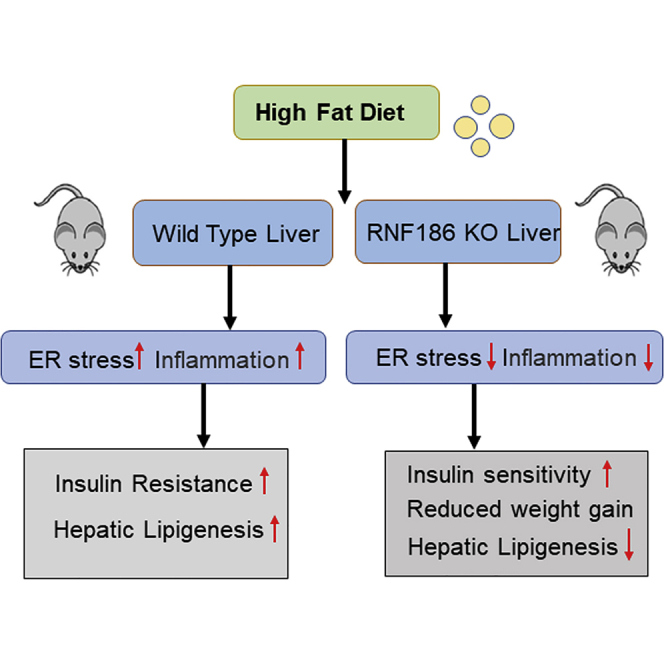

## Introduction

Non-alcoholic fatty liver disease (NAFLD) is the most common chronic liver disease globally (Ahmed et al., 2015; Cohen et al., 2011) with an incidence of approximately 25% (Younossi et al., 2016). The progression of steatosis in NAFLD ranges from simple steatosis to steatosis with inflammation (steatohepatitis), more advanced fibrosis, liver cirrhosis, and even hepatocellular carcinoma (Cohen et al., 2011; Loomba et al., 2021).The increasing morbidity rate will continue to increase economic burdens and pose a serious threat to health. Many factors lead to the development of NAFLD. These include chronic low-grade inflammation, insulin resistance, dyslipidemia, and obesity (Temple et al., 2016). Steady progress has been made in elucidating the pathogenesis of NAFLD, identifying therapeutic targets, and advancing drug development. However, the specific mechanism and effective therapeutic method remain largely unknown.

RING finger protein 186 (RNF186) is a RING-type ubiquitin E3 ligase that contains a RING finger domain (Nakamura, 2011; Wang et al., 2013). More than 200 RNF family genes have been identified. Many have diverse functions in different biological and pathological processes (Okamoto et al., 2020). Several dedicated ubiquitin ligases have critical roles in the regulation of hepatic metabolism. For example, RNF5 is important in the regulation of liver cholesterol synthesis by regulating the expression of sterol regulatory element-binding protein 2 (SREBP2) (Kuan et al., 2020). RNF20 may be involved in the regulation of triglyceride (TG) synthesis by promoting polyubiquitination and degradation of SREBP1c, leading to a decrease in the expression of lipogenic genes (Lee et al., 2014). Several lines of evidence suggest that RNF186 is involved in the pathogenesis of intestinal inflammation by participating in the regulation of ER stress (Fujimoto et al., 2017; Rivas et al., 2016). Recently, RNF186 was reported to be essential for the control of nutrient sensing through the ubiquitination of Sestrin-2, a critical cellular process controlling metabolism (Lear et al., 2019). We previously demonstrated that RNF186 impairs insulin sensitivity by inducing ER stress *in vitro* (Tong et al., 2018). However, while this enables RNF186 to regulate insulin signaling, the role of RNF186 in hepatocyte metabolism remains largely unknown.

The major goal of the current study was to explore the function of liver RNF186 in the regulation of insulin tolerance and obesity-associated NAFLD. The present findings demonstrate the involvement of RNF186 in the regulation of hepatic insulin action *in vivo*. Deletion of RNF186 alleviated insulin resistance and ER stress induced by a high-fat diet (HFD), leading to a decrease in the expression of lipogenic genes. These findings suggest that RNF186 may play an important role in liver lipid metabolism by regulating insulin signaling pathways.

## Results

### Hepatocyte RNF186-KO mice are protected against HFD-induced obesity

**Figure 1:**
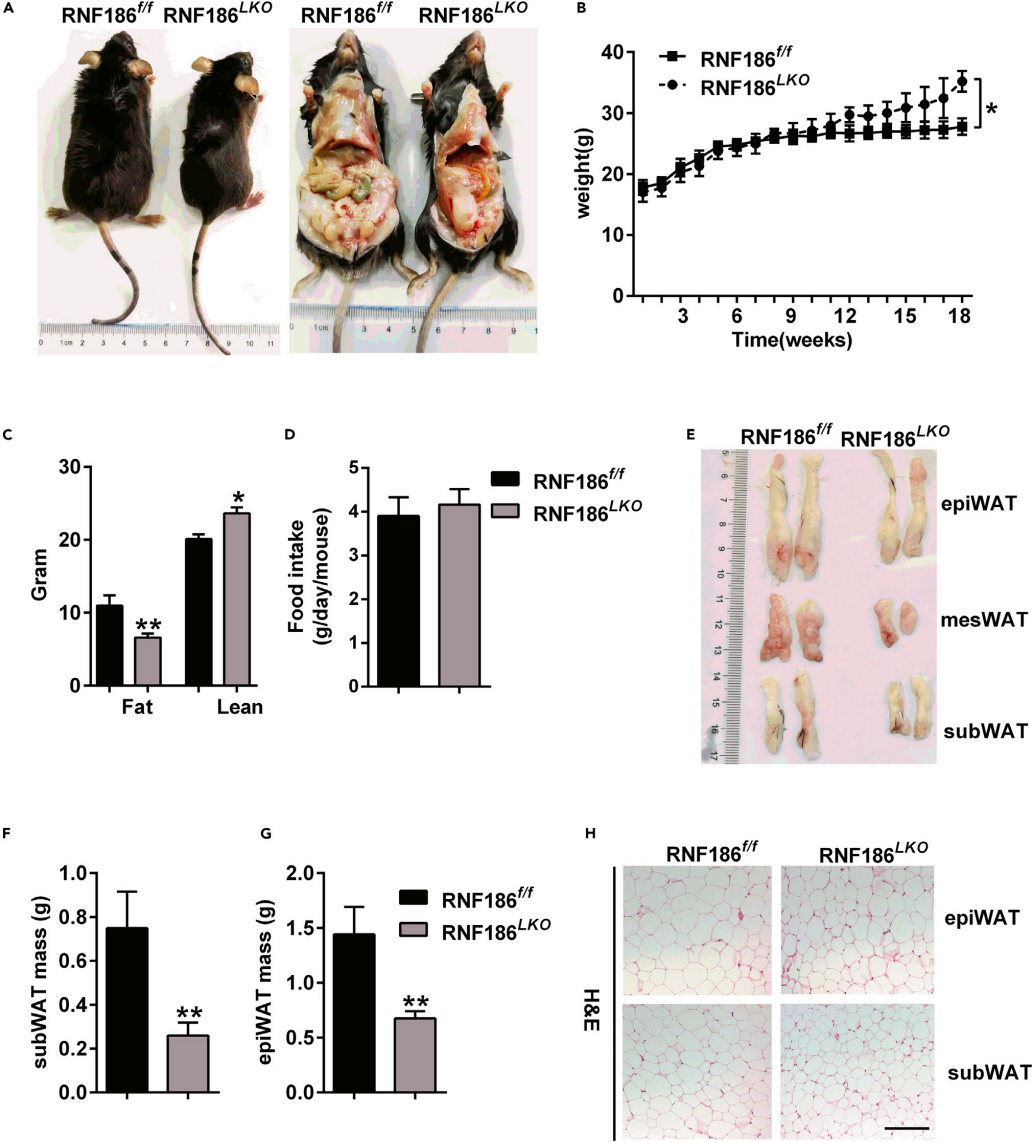
Hepatocyte RNF186 KO mice are protected from high-fat-diet (HFD)-induced obesity (A) The representative gross morphology of control and RNF186^*LKO*^ mice after 18 weeks of the HFD (n = 7 per genotype). (B) Body weights of control and RNF186^*LKO*^ mice were measured weekly from weeks 1–18 (n = 7 per genotype). (C) Fat body weight and lean body weight of control and RNF186^*LKO*^ mice at 18 weeks of age fed the HFD (n = 7 per genotype). (D) Food intake of control and RNF186^*LKO*^ mice fed with HFD (n = 7 per genotype). (E) Gross morphology of different fat tissue mass of control and RNF186^*LKO*^ mice after 18 weeks of the HFD (n = 7 per genotype). (F–G) The weight of different fat pads in control and RNF186^*LKO*^ mice (n = 7 per genotype). (H) H&E staining of epiWAT and subWAT of RNF186^*f/f*^ and RNF186^*LKO*^ mice; scare bars, 50 μm (n = 7 per genotype).Data are presented as means ± SEM ∗p < 0.05, ∗∗p < 0.01 by Student's test.

Our previous study confirmed that RNF186 expression was significantly increased in the liver tissues of HFD-induced obese mice and type 2 diabetic mice (Tong et al., 2018). Furthermore, RNF186 mediated insulin signaling pathway regulation by regulating ER stress *in vitro* (Tong et al., 2018). To further determine the specific role of RNF186 in the liver, we generated hepatocyte-specific RNF186 knockout (RNF186^*LKO*^) mice by crossing albumin-Cre mice with RNF186^*flox/flox*^ (hereafter referred to as RNF186^*f/f*^) mice. The mRNA and protein levels of RNF186 were significantly reduced in liver tissue from RNF186^*LKO*^ mice compared with control mice (Figure S1). To test the function of hepatocytes in the development of insulin resistance associated with diet-induced obesity, we fed RNF186^*f/f*^ and RNF186^*LKO*^ mice an HFD for 18 weeks. Both genotypes gained weight on the diet. However, body weight gain was less in RNF186^*LKO*^ mice compared with control mice (Figures 1A and 1B). Upon normal chow diet feeding, body weights were comparable between the genotypes (Figure S2). Additionally, RNF186^*LKO*^ mice trended toward a lower body weight, had reduced total body fat compared to control mice, and the lean mass of the RNF186^*LKO*^ mice was significantly higher than that of the control mice (Figure 1C). However, there was no difference in food intake between the RNF186^*f/f*^ and RNF186 KO mice despite the difference in weight gain (Figure 1D). We also examined white adipose tissue (WAT) of RNF186^*LKO*^ mice. The KO mice had relatively less WAT compared to the control group (Figures 1E–1G). Histological analysis revealed an increase in the occurrence of multiple lipid droplets and acidophilic adipocytes in the WAT of KO mice, especially in epididymal WAT (epiWAT) and subcutaneous WAT (subWAT) (Figure 1H).

### RNF186 deletion protects against hepatic steatosis induced by the HFD

**Figure 2:**
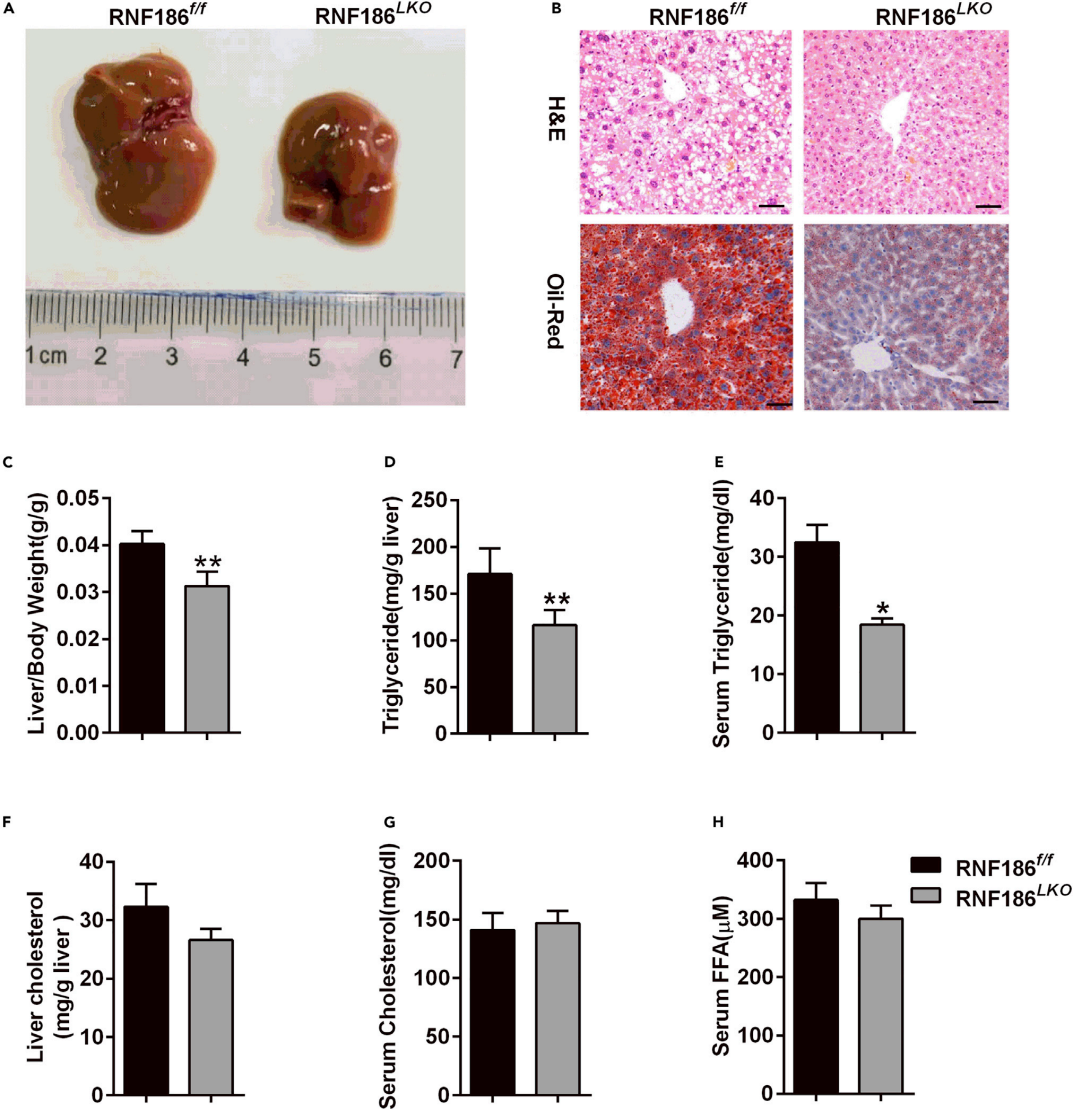
Protection against hepatic steatosis in RNF186^*LKO*^ mice (A and B) Representative results of (A) morphology, (B) H&E staining of sections (top panel), and Oil Red O staining (bottom panel) of livers from control and RNF186^*LKO*^ mice after the 18-week HFD; scare bars, 50 μm (n = 7 per genotype). (C) The ratio of the liver weight to body weight in wild type and KO mice (n = 7 per genotype). (D and E) Hepatic (D) and serum (E) TG levels in control and RNF186^*LKO*^ mice after 18 weeks of the HFD and following a 6-h fast (n = 7 per genotype). (F and G) Hepatic (F) and serum (G) TC levels in control and RNF186^*LKO*^ mice after 18 weeks of the HFD and following a 6-h fast (n = 7 per genotype). (H) Serum NEFA levels in control and RNF186^*LKO*^ mice after 18 weeks of the HFD and following a 6-h fast (n = 7 per genotype).Data are present as means ± SEM ∗p < 0.05, ∗∗p < 0.01 by Student's test.

To evaluate the effect of RNF186 on hepatic steatosis, livers were removed and observed. Compared with the control group, the ratio of liver weight to body weight of RNF186 KO mice was significantly decreased after the 18-week HFD (Figures 2A and 2C). No difference was observed in livers between two genotypes fed a standard chow diet (data not shown). Histological analysis (H&E staining) and Oil red O staining of liver sections revealed minimal lipid deposition in the livers of RNF186 KO mice compared with that in control group after 18 weeks of HFD treatment (Figure 2B). Biochemical analysis also revealed that hepatocyte-specific RNF186 KO mice significantly reduced HFD-induced accumulation of total hepatic and serum TG (Figures 2D and 2E), with comparable hepatic and serum cholesterol (TC) content and non-esterified fatty acid levels (Figures 2F–2H). However, there was no significant difference in hepatic very low-density lipoprotein (VLDL) secretion between RNF186^*LKO*^ mice and control mice (Figure S3). The collective results suggested that ablation of RNF186 in liver significantly ameliorated hepatic steatosis-induced HFD-induced obesity.

### Liver-RNF186 KO mice display improved glucose tolerance and insulin tolerance

**Figure 3:**
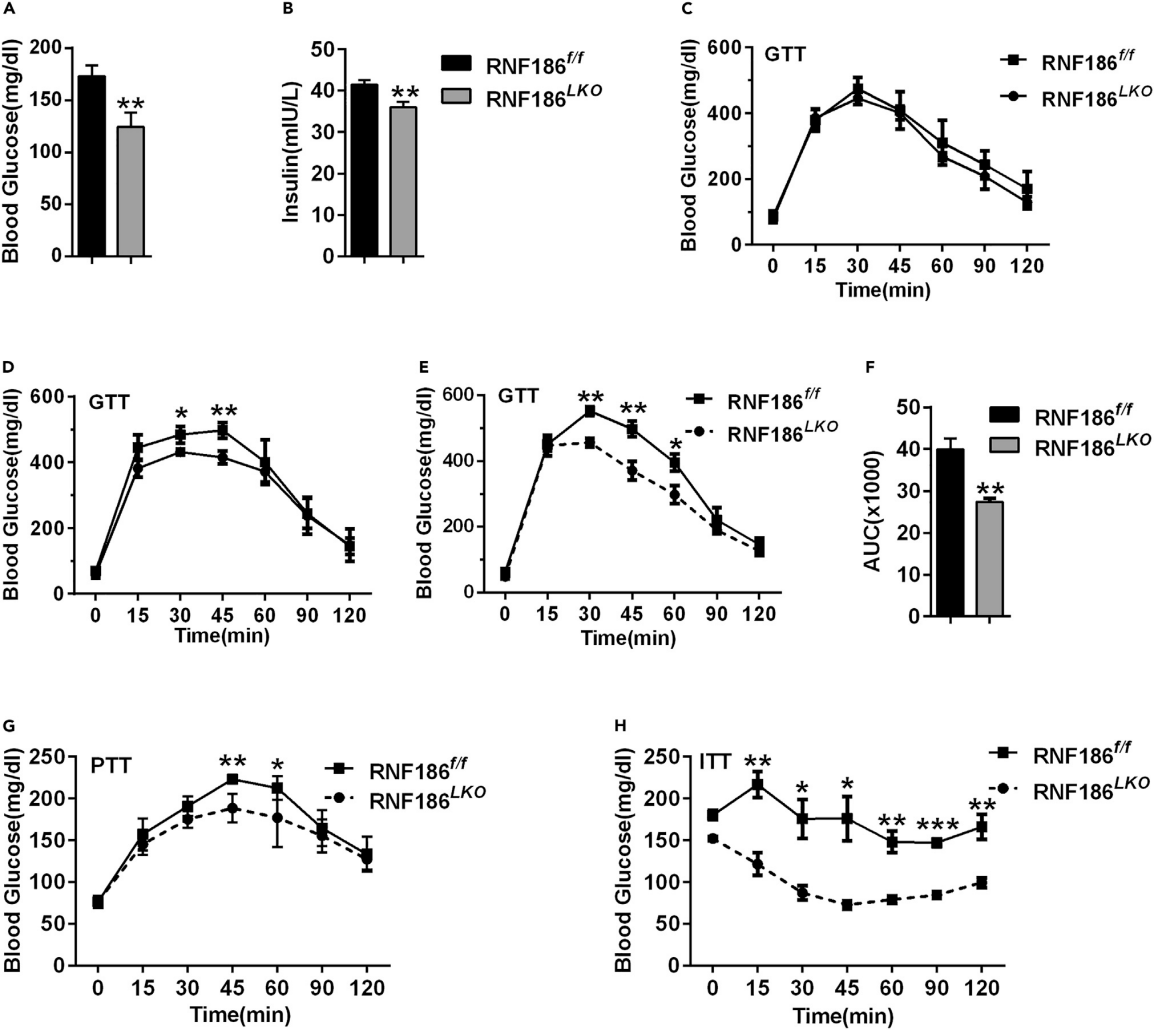
Ablation of RNF186 improves glucose tolerance and hepatic insulin tolerance (A) Blood glucose levels of control and RNF186^*LKO*^ mice after 18 weeks of the HFD followed by a 6-h fast (n = 7 per genotype). (B) Serum insulin levels for control and RNF186^*LKO*^ mice after 18 weeks of the HFD followed by a 6-h fast (n = 7 per genotype). (C–F) GTTs analyses were performed in control and KO mice after 4 (C), 8 (D), and 18 (E and F) weeks of the HFD (n = 6 per genotype). (G–H) PTTs (G) and ITTs (H) analyses were performed in control and KO mice after 18 weeks of the HFD (n = 6 per genotype).Data are present as means ± SEM ∗p < 0.05, ∗∗p < 0.01, ∗∗∗p < 0.001 by Student's test.

We next examined whether liver-specific ablation of RNF186 influenced glucose metabolism in HFD-fed mice. Fasting glucose levels and plasma insulin levels of RNF186^*LKO*^ mice were markedly lower than those of control mice fed the HFD for 18 weeks (Figures 3A and 3B). Glucose tolerance tests (GTTs) results indicated a significantly lower plasma glucose level in RNF186 KO mice compared with the control group after 8 weeks (Figures 3C and 3F). Moreover, pyruvate tolerance tests (PTTs) data confirmed that pyruvate tolerance was improved in RNF186^*LKO*^ mice compared with RNF186^*f/f*^ controls (Figure 3G). Insulin tolerance tests (ITTs) findings demonstrated that the decrease in the plasma glucose level was significantly lower in RNF186 KO mice than in control mice after insulin challenge (Figure 3H). These results suggest that liver-specific knockout of RNF186 can significantly improve glucose tolerance and insulin tolerance induced by obesity.

### Gene enrichment analyses from liver explore a comparable role of RNF186 deletion in lipid metabolism with HFD treatment

**Figure 4:**
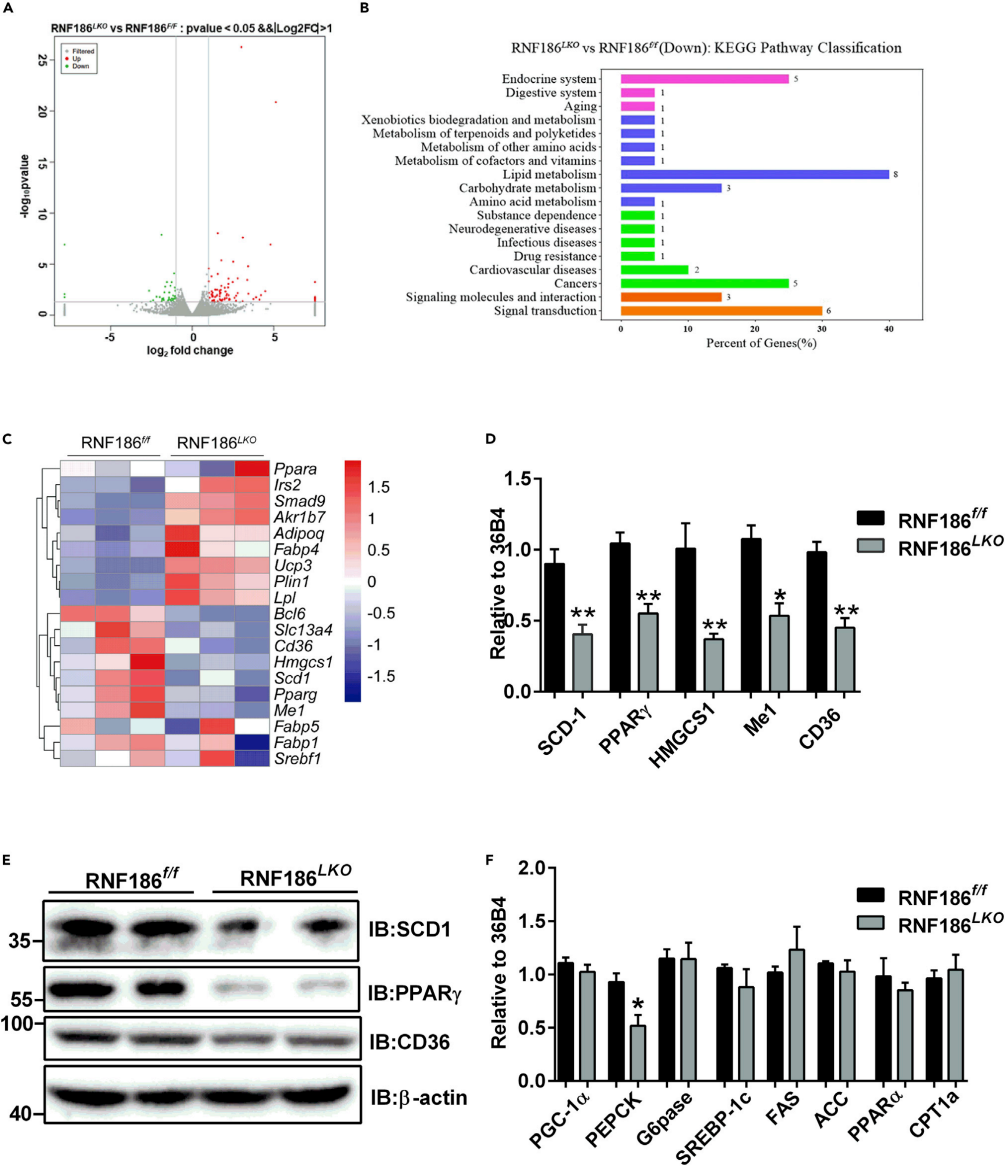
RNF186 regulates hepatic TG content in a lipogenesis-dependent manner (A) Scatterplot of differentially expressed genes of RNA-seq in livers from RNF186^*f/f*^ and RNF186^*LKO*^ mice after 18 weeks of the HFD (n = 3 per genotype). (B) KEGG pathway analysis showing downregulated pathways by RNA-seq. (C) Heatmap showing the expression of genes involved in hepatic lipid metabolism of RNA-seq (n = 3 per genotype). (D and E) qPCR (D) and western blot (E) analysis of genes involved in lipogenesis and fatty acid transport in the liver of RNF186^*f/f*^ and RNF186^*LKO*^ mice after 18 weeks of the HFD (n = 5 per genotype). (F) qPCR analysis of genes involved in gluconeogenesis, lipogenesis, and fatty acid oxidation in the livers of RNF186^*f/f*^ and RNF186^*LKO*^ mice after 18 weeks of the HFD (n = 5 per genotype).Data are present as means ± SEM ∗p < 0.05, ∗∗p < 0.01 by Student's test.

To systemically elucidate the underlying mechanism by which RNF186 affects glucose and lipid metabolism *in vivo*, we performed RNA-seq on liver tissue from RNF186^*LKO*^ and control mice. Principal component analysis separated three KO samples (red) from three control samples. A total of 21,878 genes remained for analysis after filtering out genes with little or no expression. A total of 124 genes were differentially expressed as shown in the Volcano plot in Figure 4A. Kyoto Encyclopedia of Genes and Genomes (KEGG) pathway analysis showed that deletion of RNF186 in liver significantly altered the fat metabolism and the peroxisome proliferator-activated receptor (PPAR) signaling pathway (Figure 4B).

A heatmap of the differential expression analysis revealed the gene expression signatures of the most differentially expressed genes in these samples. The heatmap summary of the results of the partial differential expression analysis revealed the expression characteristics of genes that were differentially related to lipid metabolism in these samples. Among these differential genes, those related to lipid synthesis, such as stearoyl-CoA desaturase 1 (SCD1), peroxisome proliferator-activated receptor gamma (PPARγ), and Hmgcs1, were reduced by RNF186 deficiency (Figure 4C). The findings suggested that deletion of the RNF186 gene may downregulate lipid synthesis in the liver. Consistent with the RNA-seq data, qRT-PCR results further validated the significant decreases in the expressions of SCD1, PPARγ, and HMGCS1, which are involved in the control of lipogenesis in liver of RNF186^*LKO*^ mice (Figure 4D). Western blot analyses revealed the reduced protein levels of SCD1 and PPARγ in the livers of RNF186^*LKO*^ mice compared with control mice (Figure 4E). We also identified the decreased mRNA and protein levels of cluster of differentiation 36 (CD36), the fatty acid translocase protein, in hepatic tissue of RNF186 KO mice compared with the control groups (Figures 4D and 4E). Consistent with the decreased blood glucose levels, the expression of gluconeogenic genes and phosphoenolpyruvate carboxykinase (PEPCK) was decreased in the livers of RNF186^*LKO*^ mice (Figure 4F). However, the mRNA levels of SREBP-1c and its target genes, including fatty acid synthase and acetyl-CoA carboxylase, were unchanged (Figure 4F). Additionally, there were not obvious alterations of the activity of genes related to fatty acid oxidation (Figure 4F). These data suggest that deletion of RNF186 can downregulate lipogenic gene expression due to HFD-induced obesity and protect against liver steatosis.

### Deletion of RNF186 in liver alleviates insulin signaling in diet-induced obesity

**Figure 5:**
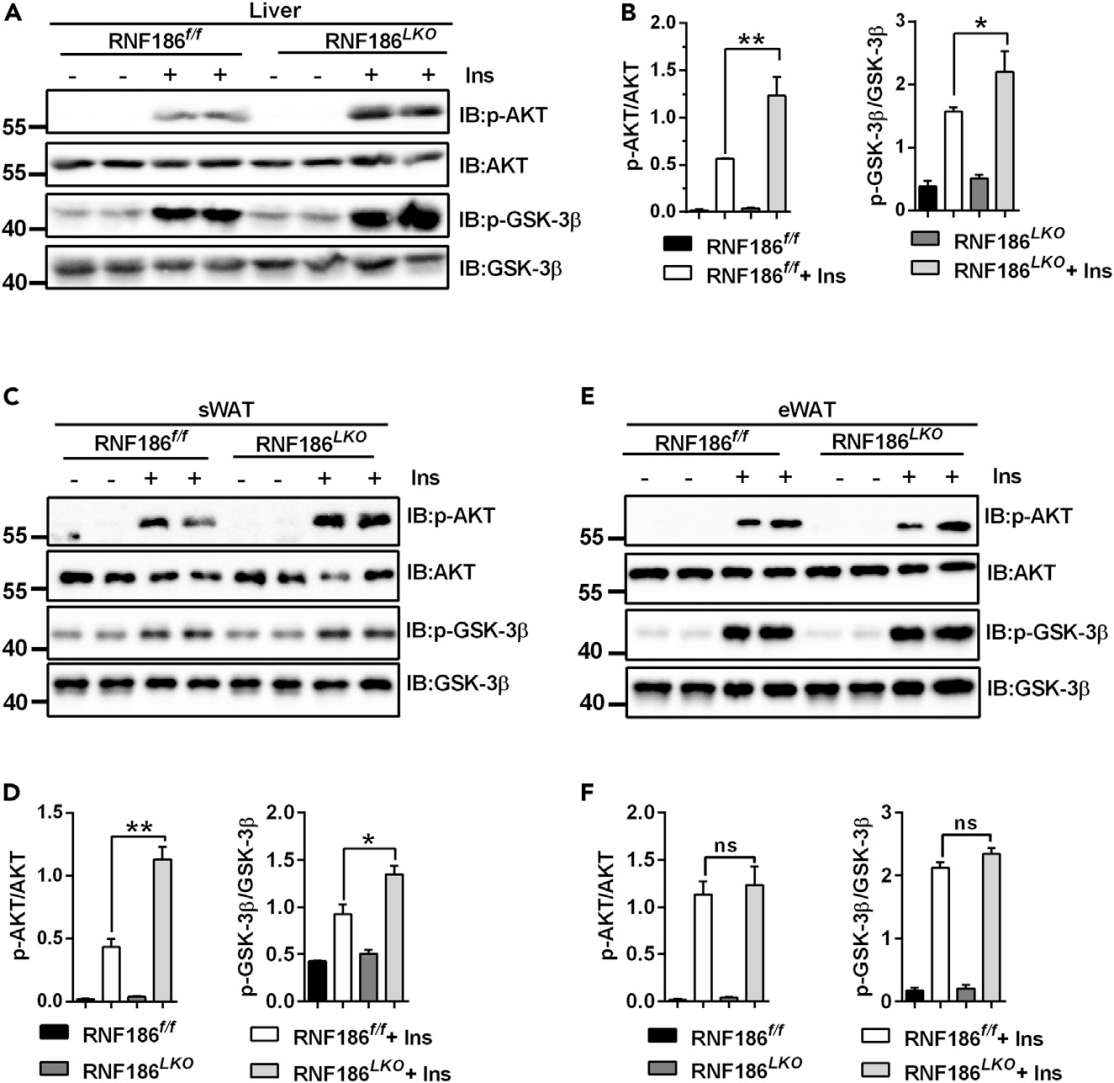
RNF186 deficiency ameliorates insulin tolerance induced by HFD (A) Western blot analysis of insulin-stimulated AKT and GSK-3β phosphorylation in liver of RNF186^*f/f*^ and RNF186^*LKO*^ mice after 18 weeks of the HFD (n = 4 per genotype). (B) Quantitation of the western blot data shown in (A). (C) Western blot analysis of insulin-stimulated AKT and GSK-3β phosphorylation in subWAT of RNF186^*f/f*^ and RNF186^*LKO*^ mice after 18 weeks of the HFD (n = 4 per genotype). (D) Quantitation of the western blot data shown in (C). (E) Western blot analysis of insulin-stimulated AKT and GSK-3β phosphorylation in epiWAT of RNF186^*f/f*^ and RNF186^*LKO*^ mice after 18 weeks of the HFD (n = 4 per genotype). (F) Quantitation of the western blot data shown in (E).Data are present as means ± SEM ns > 0.05, ∗p < 0.05, ∗∗p < 0.01 by one-way ANOVA.

Our previous studies confirmed that RNF186 can regulate insulin sensitivity *in vitro* (Tong et al., 2018). Therefore, in the present study, we further investigated the effect of RNF186 on insulin signaling *in vivo*. We injected insulin or saline into obese mice of different genotypes through the inferior vena cava after a 16-h fast and quickly removed the liver and adipose tissue to analyze the insulin signaling pathway. The results confirmed that the phosphorylation levels of AKT increased in the liver RNF186 KO mice fed the HFD relative to control mice (Figures 5A and 5B). Consistent with this result, glycogen synthase kinase 3 beta (GSK-3β) phosphorylation was increased in the liver of RNF186^*LKO*^ mice compared with control mice (Figures 5A and 5B). We also observed that subWAT (Figures 5C and 5D), but not epiWAT (Figures 5E and 5F), of RNF186 KO mice showed increased insulin-induced AKT and GSK-3β phosphorylation.

### RNF186 deficiency protects mice from HFD-induced liver inflammation

**Figure 6:**
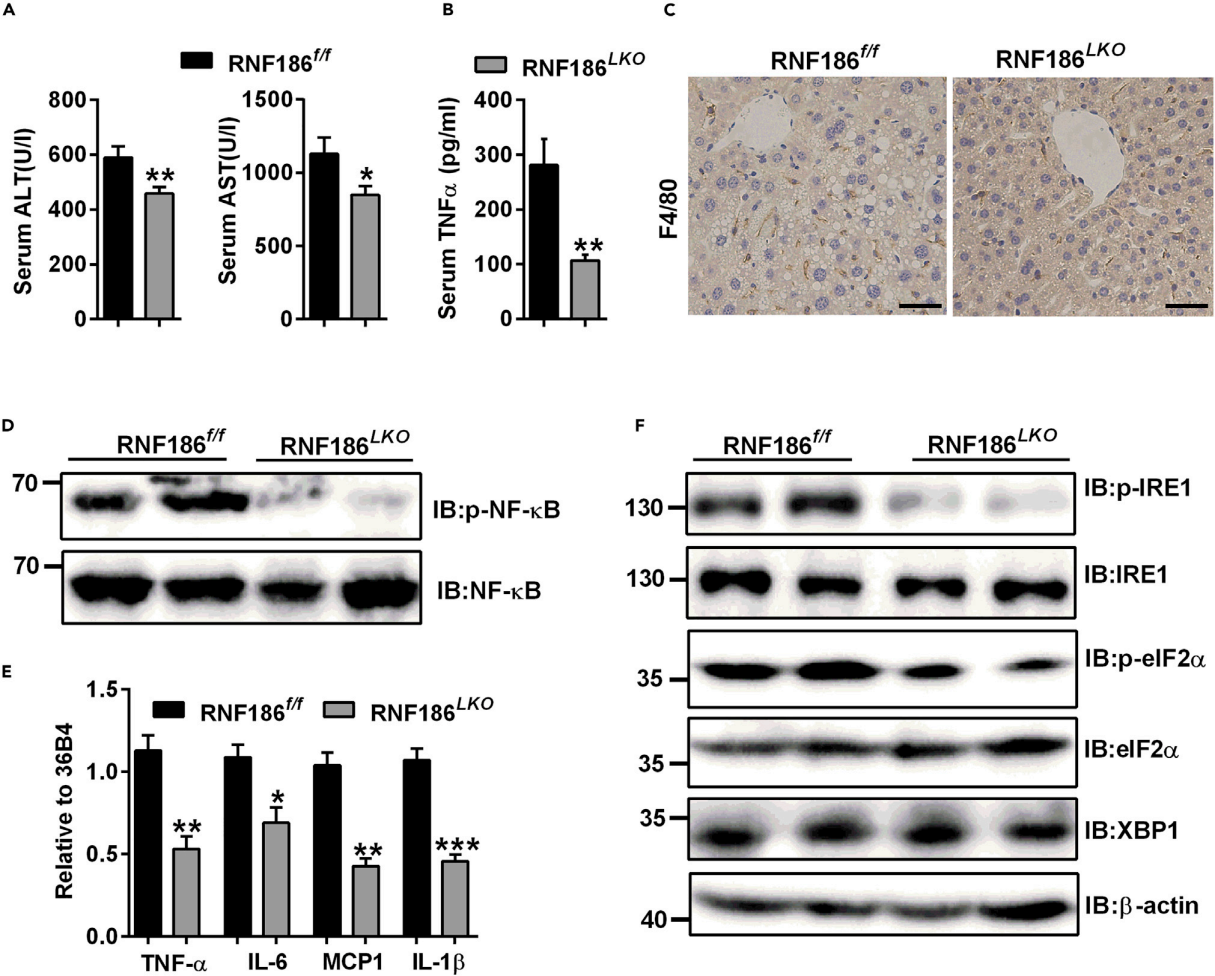
RNF186 deficiency protects mice from HFD-induced hepatic inflammation (A) Hepatic deletion RNF186 decreased serum ALT and AST levels (n = 7 per genotype). (B) Serum TNFα levels for control and RNF186^*LKO*^ mice after 18 weeks of the HFD followed by a 6-h fast (n = 7 per genotype). (C) Histological F4/80 staining in liver sections from RNF186^*f/f*^ and RNF186^*LKO*^ mice fed the HFD for 18 weeks; scare bars, 25 μm (n = 4 per genotype). (D) Western blot analysis of NF-κB protein and phosphorylation levels in liver of RNF186^*f/f*^ and RNF186^*LKO*^ mice after 18 weeks of the HFD (n = 5 per genotype). (E) qPCR analysis of genes involved in inflammation response in the livers of wild-type and RNF186^*LKO*^ mice fed the HFD for 18 weeks (n = 5 per genotype). (F) Western blot analysis of the ER stress marker proteins IRE1 and eIF2α phosphorylation levels in livers of RNF186^*f/f*^ and RNF186^*LKO*^ mice after 18 weeks of the HFD (n = 5 per genotype).Data are present as means ± SEM ∗p < 0.05, ∗∗p < 0.01, ∗∗∗p < 0.001 by Student's test.

Because HFD-induced obesity is associated with the development of inflammation, we further analyzed whether RNF186^*LKO*^ can alleviate inflammation induced by an HFD. RNF186 KO mice displayed lower levels of serum alanine transaminase (ALT) and aspartate transaminase (AST) in serum than the levels in the control group after consumption of the HFD (Figure 6A). We also measured serum concentrations of tumor necrosis factor -α (TNFα), which has postulated roles in obesity and insulin action (Khan et al., 2019). RNF186 KO mice displayed lower serum TNFα levels than in control mice (Figure 6B). In addition, we performed immunohistochemical analysis of liver sections from two genotypes mice, using an antibody specific for F4/80 antigen, a pan-macrophage marker. The number of F4/80 positive cells was markedly decreased in the livers of RNF186 KO mice compared with control group (Figure 6C). Western blot analysis confirmed that the phosphorylation levels of nuclear factor kappa B (NF-κB) was reduced in the livers of RNF186^*LKO*^ mice compared to the levels in control mice (Figure 6D). Consistent with this, downregulation of TNFα, interleukin-6 (IL-6), IL-1β, and monocyte chemoattractant protein-1 (MCP1) due to RNF186 deficiency was identified by RT-PCR in liver tissue (Figure 6E). Our previous study demonstrated that overexpression of RNF186 induces ER stress and increases inflammation *in vitro* (Tong et al., 2018). Here, deletion of RNF186 decreased inositol-requiring kinase 1 (IRE1) and eukaryotic initiation factor 2α (eIF2α) phosphorylation levels in the livers of mice fed the HFD compared with control mice (Figure 6F). However, the protein level of X-box binding protein 1 (XBP-1s) showed no change between two groups (Figure 6F). These results suggest that knockout of RNF186 in the liver may have a protective effect on HFD-induced inflammation.

### Overexpression of RNF186 promotes TG accumulation and inflammation in the liver of C57BL/6J mice

**Figure 7:**
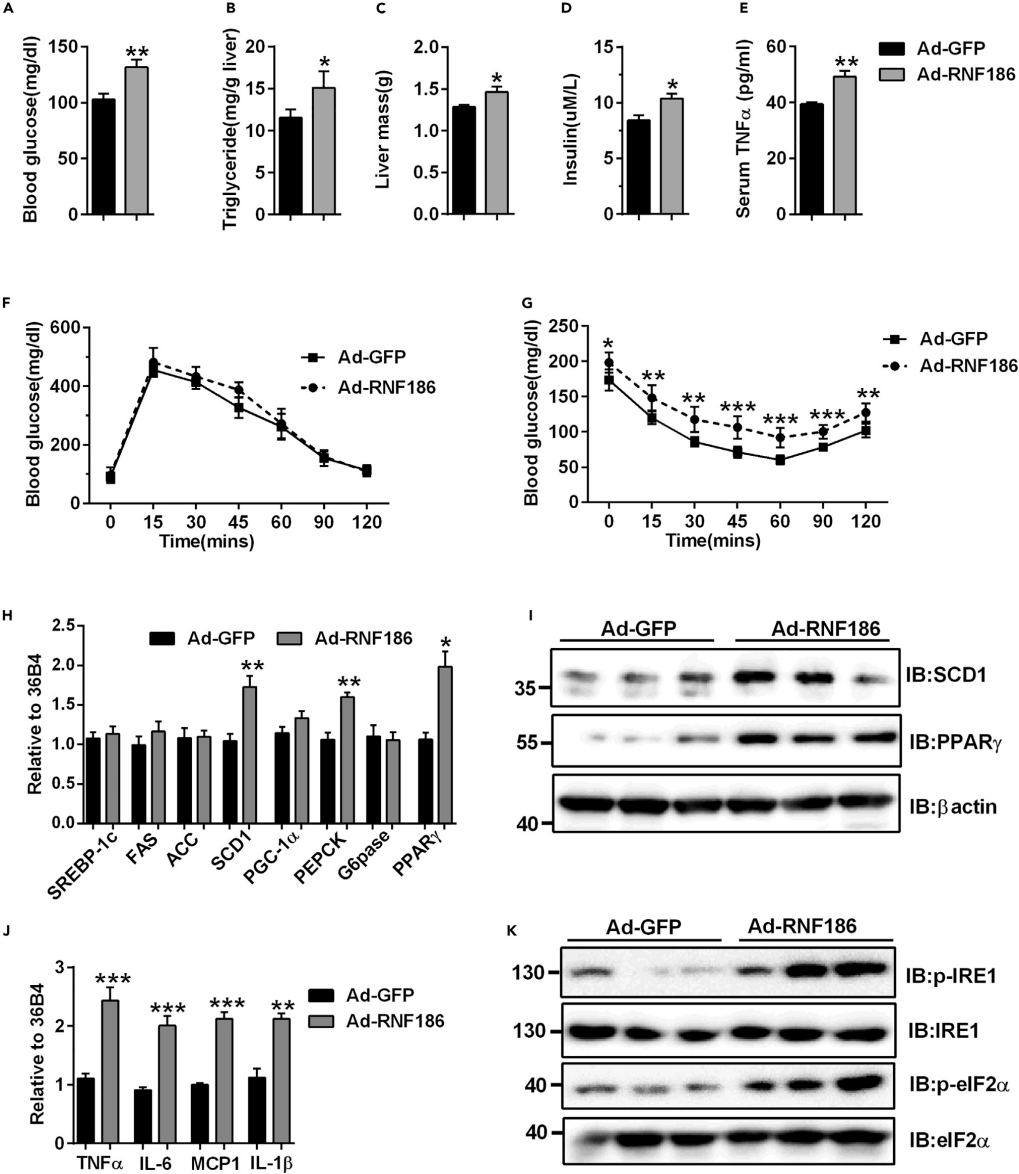
Overexpression of RNF186 in the liver of C57BL/6J mice exacerbates lipid metabolism (A) Blood glucose level in control C57BL/6J mice injected with Ad-GFP or Ad-RNF186 9 days after injection under fasting conditions (n = 7 per group). (B) Change of hepatic TG levels in C57BL/6J mice infected with Ad-GFP or Ad-RNF186 after 9 days under fasting conditions (n = 7 per group). (C) Hepatic RNF186 overexpression significantly increased the liver weight in mice infected with Ad-RNF186 compared with the control mice. (D and E) Change of serum insulin (D) and TNFα (E) levels in C57BL/6J mice infected with Ad-GFP or Ad-RNF186 under fasting conditions (n = 7 per group) (F and G) GTTs (F) and ITTs (F) in control C57BL/6J mice injected with Ad-GFP or Ad-RNF186 5 days after injection (n = 5 per group). (H) Relative mRNA levels of genes involved in lipogenesis and gluconeogenesis in the liver of C57BL/6J mice infected with Ad-GFP or Ad-RNF186 (n = 5 per group). (I) Western-blot analysis of PPARγ and SCD1 in the liver of C57BL/6J mice infected with Ad-GFP or Ad-RNF186 (n = 5 per group). (J) qPCR analysis of genes involved in inflammation response in the liver of C57BL/6J mice infected with Ad-GFP or Ad-RNF186 (n = 5 per group).(I) Western-blot analysis of PPARγ and SCD1 in the liver of C57BL/6J mice infected with Ad-GFP or Ad-RNF186 (n = 5 per group). (K) IRE1 and eIF2α phosphorylation were analyzed by western blot in the liver of C57BL/6J mice infected with Ad-GFP or Ad-RNF186 (n = 5 per group).Data are present as means ± SEM ∗p < 0.05, ∗∗p < 0.01, ∗∗∗p < 0.001 by Student's test.

To further explore the role of RNF186 in the progression of NAFLD, we upregulated RNF186 expression in the liver of C57BL/6J mice by tail vein injection of Ad-RNF186. qPCR and western blots showed that adenovirus-mediated RNF186 was effectively expressed in the liver compared with control adenovirus Ad-GFP-treated mice (Figures S4A and S4B). Overexpression of RNF186 increased the fasting glucose levels compared with control groups (Figure 7A). Meanwhile, overexpression of RNF186 led to a markedly upregulation in hepatic TG content in C57BL/6J mice (Figure 7B), whereas serum or hepatic total TC and FFAs were comparable between two groups (data not shown). The liver weight was obviously higher in RNF186 overexpression mice than in control mice (Figure 7C). Furthermore, we found that Ad-RNF186-infected mice significantly increased the serum insulin and TNFα levels compared with the levels in control group (Figures 7D and 7E). GTTs and ITTs indicated that hepatic overexpression of RNF186 had no effect on glucose tolerance and impaired insulin tolerance compared with the control mice (Figures 7F and 7G). Additionally, the mRNA expression of SCD1 and PPARγ was significantly higher in mice infected with Ad-RNF186 than in mice infected with Ad-GFP (Figures 7H and 7I). We also observed the expression of mRNA proinflammatory factors genes, including TNFα, IL6, and MCP1, was increased in the livers of mice infected with Ad-RNF186 compared with those of control mice. Our previous study has been reported that overexpression of RNF186 induced ER stress and increased the phosphorylation levels of IRE1 and eIF2α in primary hepatocytes (Tong et al., 2018). Consistently, the phosphorylation of IRE1 and eIF2α was significantly higher in the liver of mice infected with Ad-RNF186 than the control mice (Figure 7K). These results indicate that upregulation of RNF186 impairs lipid metabolism and induced inflammation in the liver of C57BL/6J mice.

## Discussion

It has long been known that the common feature of NAFLD is insulin resistance. Insulin resistance increases the levels of insulin in the serum, which causes hepatocytes to promote hepatic lipid synthesis through several insulin-sensitive signaling factors and induces a vicious cycle of inflammation (Farese et al., 2012; Tilg and Moschen, 2008). Growing evidence indicates that adipose tissue dysfunction/inflammation is crucial in NAFLD pathogenesis. Studies have revealed the metabolic crosstalk between adipose tissue and the liver.

Previously, we reported that the protein expression of RNF186 was upregulated in the fatty liver compared with the control group and that RNF186 impairs insulin sensitivity by increasing ER stress and promotes TG production from primary hepatocytes (Tong et al., 2018). However, the precise molecular mechanisms of RNF186 in the regulation of hepatic fat metabolism *in vivo* have not been established. In the present study, we demonstrate that RNF186 is crucial in exacerbating HFD-induced lipid accumulation, inflammation, and insulin resistance in hepatocytes. Hepatocyte-specific RNF186 KO mice were protected from hepatic insulin resistance induced by HFD feeding for 18 weeks. It is manifested as an increase in the phosphorylation level of AKT in the liver of RNF186-KO mice. In addition, we also found a significant improvement in pyruvate tolerance in KO mice, and pyruvate serves as a major substrate for hepatic gluconeogenesis (Wang et al., 2009), so we speculated that RNF186 KO mice had a significant improvement in hepatic gluconeogenesis compared with the control mice. In addition, we found that RNF186*LKO* mice protected against HFD-induced obesity, but there was no difference in food intake between KO mice and control mice. The mechanism is still unclear and needs further study. We hypothesized that liver-specific knockout of RNF186 might cause changes in adipose tissue energy consumption.

The RNF family is involved in the regulation of hepatic lipid metabolism through different mechanisms. The function of RNF20 is reportedly related to hepatic lipid metabolism through the degradation of SREBP1c (Lee et al., 2014). However, RNF5 activates SREBP2 to promote cholesterol biosynthesis (Kuan et al., 2020). Herein, we demonstrate that the deletion of RNF186 leads to downregulation of the expressions of hepatic SCD1, PPARγ, and HMGCS1. SCD1 and PPARγ are important regulators of hepatic *de novo* lipogenesis and TG synthesis *in vitro* and *in vivo* (Mendez-Sanchez et al., 2007). Hepatic overexpression of PPAR-γ2 using adenovirus was sufficient to increase liver TG, along with an increase in mRNA levels of SREBP-1c and other lipogenic genes (Yamazaki et al., 2011). To address the mechanism underlying the RNF186-mediated decreases of SCD1 and PPARγ, we examined the interactions between RNF186 and these proteins in HepG2 cells. There was no evidence of these interactions. Therefore, the mechanism by which RNF186 controls these proteins remains unclear and needs to be further investigated. Additionally, the expression of the CD36 fatty acid translocase protein was downregulated in the livers of RNF186 KO mice. A previous study showed that mice fed an HFD displayed induced hepatic steatosis and increased expression of CD36 (Wilson et al., 2016). These events could contribute to the reduced lipid accretion in the livers of RNF186 KO mice.

Insulin is the master regulator of hepatic glucose and lipid metabolism through direct and indirect mechanisms. Interactions among insulin resistance, aberrant lipid metabolism, and hepatic inflammation collaboratively promote NAFLD progression (Tilg and Moschen, 2008; Wang et al., 2016). Insulin resistance is accompanied by decreases in glycogen synthesis and gluconeogenesis inhibition, which will lead to an increased release of glucose (Jornayvaz and Shulman, 2012). The present findings demonstrate that the phosphorylation levels of key insulin signaling molecules, such as AKT and GSK3β, in the liver and subWAT tissue of RNF186^*LKO*^ group were much higher than in control mice after consumption of the HFD.

Inflammatory pathways have been closely related to insulin sensitivity and glucose metabolism. In addition, the activation of the NF-κB transcription factor impairs the homeostasis of hepatocytes and increases hepatic inflammation (Chan et al., 2017). NF-κB expression is exceedingly high in liver and adipose tissue in states of insulin resistance. Inhibiting or blocking the NF-κB signaling pathway protects mice from obesity-induced insulin resistance (Dong et al., 2020; Kang et al., 2011; Ye et al., 2019). Compared with control mice, plasma ALT and AST levels were significantly decreased in RNF186 KO mice. Deletion of RNF186 alleviated the inflammatory response by decreasing NF-κB phosphorylation and decreasing the levels of intrahepatic TNFα, IL-6, and MCP1. Substantial evidence has identified many mechanisms by which activation of the unfolded protein response could differentially upregulate inflammatory pathways (Imbernon et al., 2016). Our previous study demonstrated the involvement of RNF186 in the regulation of ER stress *in vitro* (Tong et al., 2018). In fact, RNF186 deletion alleviated hepatic ER stress. Therefore, these results indicate that the deletion of RNF186 can reduce obesity-related hepatic inflammation and ER stress. In addition, our data showed that forced expression of RNF186 in the livers of C57BL/6J mice increased the hepatic TG levels and impaired lipid metabolism and insulin sensitivity. However, we should note that in the present study, we did not find the direct substrate of RNF186. Further studies are necessary to explore the precise mechanism of how RNF186 regulates lipid metabolism and insulin action *in vivo*.

In conclusion, RNF186 deficiency in liver protected against obesity and in turn liver steatosis, insulin resistance, and hepatic inflammation induced by an HFD. Our results implicate hepatocyte RNF186 may be as a novel modulator of lipid metabolism in the liver.

### Limitations of the study

Altogether, RNF186 KO mice are protected against HFD-induced weight gain and associated fatty liver, insulin resistance, and hyperglycemia. These results may suggest a direct role, but are confounded by the differences in body weight in the models presented and will require further study. It is possible that liver-specific knockout of RNF186 may affect the secretion of related factors in mouse liver and act on adipose tissue, and regulate the accumulation of triglycerides in adipose tissue or affect energy expenditure. In the future, we will further explore the role of RNF186 in the metabolism of adipose tissue by constructing adipose tissue-specific RNF186 knockout mice.

## STAR★Methods

### Key resources table

**Table:** 

REAGENT or RESOURCE	SOURCE	IDENTIFIER
**Antibodies**		

Rabbit anti-SCD-1	ABclonal	Cat# A16429; RRID: AB_2772150
Rabbit anti-PPARγ	Cell Signaling Technology	Cat# 2435T; RRID: AB_2166051
Rabbit anti-CD36	Abclonal	Cat# A5792; RRID: AB_2766544
Rabbit anti-β-actin	Cell Signaling Technology	Cat# 4970S; RRID: AB_2223172
Rabbit anti-AKT	Cell Signaling Technology	Cat# 9272S; RRID: AB_329827
Rabbit anti-p-AKT	Cell Signaling Technology	Cat# 9271S; RRID: AB_329825
Rabbit anti-GSK-3β	Cell Signaling Technology	Cat# 9315S; RRID: AB_490890
Rabbit anti-p-GSK-3β	Cell Signaling Technology	Cat# 5558S; RRID: AB_10013750
Rabbit anti-NF-κB	Cell Signaling Technology	Cat# 8242T; RRID: AB_10859369
Rabbit anti-p-NF-κB	Cell Signaling Technology	Cat# 3033T; RRID: AB_331284
Rabbit anti-IRE1α	Cell Signaling Technology	Cat# 3294T; RRID: AB_823545
Rabbit anti-p-IRE1α	ABclonal	Cat# AP0878; RRID: AB_2771207
Rabbit anti-eIF-2α	Cell Signaling Technology	Cat# 5324T; RRID: AB_10692650
Rabbit anti-p-eIF-2α	ABclonal	Cat# 3398T; RRID: AB_2096481
Rabbit anti-XBP1	BOSTER	Cat# PB9463
Rabbit anti-RNF186	Sangon	Cat# YS-2692R
Rabbit anti-F4/80	Abcam	Cat# ab6640; RRID: AB_1140040

**Bacterial and virus strains**		

Ad-GFP	Tong et al. (2018)	https://doi.org/10.1016/j.cellsig.2018.09.008
Ad-RNF186	Tong et al. (2018)	https://doi.org/10.1016/j.cellsig.2018.09.008

**Chemicals, peptides, and recombinant proteins**		

Insulin	Novo Nordisk	Cat# 8-0212-03
PVDF membranes	Millipore	Cat# IPVH00010
Oil Red O	Solarbio	Cat# O8010
Sodium Pyruvate	Sigma-Aldrich	Cat# P2256
Mouse Insulin ELISA Kit	Lengton Bioscience	Cat# BPE20352
Mouse TNFα ELISA Kit	R&D Systems	Cat# MHSTA50
TRizol reagent	Life Technologies	Cat# 15596026
BSA	Solarbio	Cat# A8850
iScript Reverse Transcription mix	Thermo Fisher	Cat# K1622
iQ SYBR Green Supermix	Promega	Cat# A600A
Phosphatase inhibitor cocktail	Bestbio	Cat# 33110A
Protease inhibitor cocktail	Beyotime	Cat# ST506-2
Tyloxapol	Sigma-Aldrich	Cat# 25301-02-4
Formalin	Servicebio	Cat# G1101
Cholesterol reagent	Applygen	Cat# E1015
Triglyceride reagent	Applygen	Cat# E1013
Free fatty acid reagent	Sigma-Aldrich	Cat# O7501, P9767
Alanine transaminase reagent	Roche	Cat# 05850797190
Aspartate transaminase reagent	Roche	Cat# 05850819190

**Deposited data**		

RNA-seq data of RNF186 KO and wild-type were fed HFD	This paper	SRA: PRJNA784646

**Experimental models: Organisms/strains**		

Mouse: C57BL/6J	Gempharmatech Co., Ltd	N/A
Mouse: RNF186^*flox/flox*^	Ji et al. (2020)	https://doi.org/10.1016/j.cellsig.2020.109764
Mouse: albumin-Cre mice	Nanjing Biomedical Research Institute of Nanjing University	Cat# J003547

**Oligonucleotides**		

Primer sequences for RT-qPCR	Table S1	N/A
Primer sequences for molecular cloning	Ji et al. (2020)	https://doi.org/10.1016/j.cellsig.2020.109764

**Software and algorithms**		

GraphPad Prism 6	Graphpad	http://www.graphpad.com
Photoshop	Adobe	https://www.adobe.com
Image J	NIH	https://imagej.nih.gov

**Other**		

High fat diet	SYSE bio	Cat#PD6001
Normal chow diet	XIETONG SHENGWU	Cat#1010086
OLYMPUS-IX73P1F	OLYMPUS microsystems	N/A

### Resource availability

#### Lead contact

Further information and requests for resources and reagents should be directed to and will be fulfilled by the lead contact, Huabing Zhang (slzhang1977@163.com & huabingzhang@ahmu.edu.cn).

#### Materials availability

This study did not generate new unique reagents.

### Experimental model and subject details

#### Mouse models

Liver specific RNF186 knockout (KO) mice were created by breeding floxed mice (Nanjing Biomedical Research Institute of Nanjing University, Nanjing, China). RNF186 flox/flox mice (RNF186^*f/f*^) were created using the clustered regularly interspaced short palindromic repeats (CRISPR)/CRISPR-associated 9 methods (Ji et al., 2020). These mice were then crossed with albumin-Cre mice to generate liver specific RNF186 knockout (RNF186^*LKO*^) mice. The KO efficiency was confirmed in liver tissue and other tissues by western blot analysis using anti-RNF186 antibodies. Unless otherwise noted, 8-week-old male mice were used for all experiments. All animal experiments conformed to the guidelines of the Animal Center of Anhui Medical University. The experimental procedures were approved by the Institutional Animal Care and Use Committee of Anhui Medical University.

#### Mouse experiments

Five-week-old male WT and RNF186^*LKO*^ mice were fed either standard chow diet (9% fat; Lab Diet) or HFD (45% fat; Research Diets) *ad libitum* for 18 weeks, with free access to water. C57BL/6J mice were purchased from GemPharmatech Co., Ltd (Nanjing, China). Mice were housed and maintained in a 12-h light/12-h dark cycle clean animal facility at Anhui Medical University. For adenovirus treatment, eight-week-old male C57BL/6J mice were injected with purified adenovirus with 1.0 × 10^9^ active viral particles in 150μl of 0.4% NaCl solution via tail vein. Then, 7-9 days later, mice fasted for 6 h were sacrificed, and livers and plasma were collected for analysis. Body weight and fasting blood glucose levels were examined.

### Method details

#### *In vivo* glucose, insulin, and pyruvate tolerance tests

WT and RNF186^*LKO*^ mice were fed HFD (45% fat; Research Diets) *ad libitum* for 18 weeks. For the glucose tolerance tests (GTTs) and pyruvate tolerance tests (PTTs), mice were injected with D-glucose (1–2 g/kg body weight) or pyruvate sodium (1–2 g/kg body weight) via intraperitoneal injection after 16 h of fasting. For the insulin tolerance tests (ITTs), mice were injected with insulin (0.5–0.75 U/kg body weight) via intraperitoneal injection after 6 h (from 10:00 a.m. to 4:00 p.m.) of fasting. Blood glucose levels were tested from the tail vein with a glucometer (One Touch Ultra, LifeScan Inc.) at indicated times (0, 15, 30, 45, 60, 90, 120 min after glucose, pyruvate or insulin).

#### *In vivo* insulin signaling

After an overnight fasting, mice were anesthetized with 2, 2, 2- tribromoethanol in PBS (Avertin) and injected with 5 U of regular human insulin (Novo Nordisk) via the inferior vena cava injection. Five or ten minutes after the insulin bolus, livers were removed and frozen in liquid nitrogen. Immunoblot analysis of insulin signaling molecules was performed using tissue homogenates prepared in a tissue homogenization buffer supplemented with the complete protease inhibitor cocktail (Beyotime) and Phosphatase inhibitor cocktail (Bestbio).

#### Preparation of expressing RNF186 recombinant adenoviruses

Recombinant overexpression of RNF186 adenoviruses were generated according to the manufacturer’s instructions (Invitrogen) and purified by the cesium chloride method as previously described (Tong et al., 2018).

#### RNA isolation and quantitative RT-PCR

Total RNA was extracted from tissues using a TRIzol-based method (Life Technologies, Carlsbad, California, USA). One microgram of total RNA was reverse transcribed using the High Capacity cDNA Reverse Transcription Kit and random primers according to the manufacturer’s instructions (Thermo Fisher, Waltham, MA, USA). Quantitative PCR (qPCR) was performed using the SYBR Green I qPCR kit (Promega, Madison, WI, USA) on a CFX system (Bio-Rad, Hercules, CA, USA). Gene expression data was normalized to 36B4. The specific primer sequences used for real-time PCR are listed in Table S1.

#### Western blotting

Whole tissue lysate was prepared with RIPA buffer supplemented with phosphatase inhibitor and protease inhibitor cocktail before the experiment. Western blotting was performed by utilizing a standard protocol as described previously [23]. The antibodies were anti-β-actin, anti-PPARγ, anti- NF-κB/phospho-NF-κB, anti- eIF2α/phospho-eIF2α, anti-AKT/phospho-AKT (Ser-473), anti- GSK-3β/phosphor-GSK-3β (Cell Signaling Technology, Beverly, MA, USA), anti- SCD1, anti-CD36 (ABclonal Technology, Woburn, MA, USA) and anti-RNF186 (Sangon Biotech, Shanghai, China).

#### RNA-sequencing (RNA-seq) and bioinformatics analysis

Transcriptome sequencing and analyses were conducted by OE Biotech Co. Ltd. (Shanghai, China). Briefly, Total RNA was extracted using the mirVana miRNA Isolation Kit (Ambion, Austin, TX, USA) following the manufacturer’s protocol. RNA integrity was evaluated using a model2100 Bioanalyzer (Agilent Technologies, Santa Clara, CA, USA). The samples with RNA Integrity Number (RIN) ≥ 7 were analyzed. The libraries were constructed using TruSeq Stranded mRNA LTSample Prep Kit (Illumina, San Diego, CA, USA) according to the manufacturer’s instructions. The libraries were sequenced on an Illumina sequencing platform (HiSeq 2500 or HiSeq X Ten) and 125bp/150bp paired-end reads were generated. The RNA-sequencing data have been deposited in NCBI under SRA accession numbers (SRA: PRJNA784646).

#### Biochemical analysis and cytokine measurement

Serum ALT, AST, TG, TC, and free fatty acid levels were determined in an automated device (Monach) in the clinical laboratory of the First Affiliated Hospital of Anhui Medical University, Hefei, China. Hepatic TG and TC contents were measured using a colorimetric diagnostic kit (Applygen Technologies, Inc., Beijing, China). Serum TNFα concentrations were determined by ELISA (R&D Systems, Minneapolis, MN, USA). Serum insulin concentrations were determined by ELISA (Lengton Bioscience Co.LTD, Shanghai, China).

#### Histology and immunohistochemistry

For hematoxylin and eosin (H&E) staining, liver tissue was fixed in 10% neutral-buffered formalin, embedded in paraffin, and cut into 6 μm sections. The sections were stained with H&E. For Oil red O staining, liver tissue was frozen in liquid nitrogen and cut into 10 μm sections. Specimens were stained and microscopically evaluated at 100× magnification. Immunohistochemical examination was carried out to detect the expression of F4/80 (Abcam; ab6640) in liver tissues.

### Quantification and statistical analysis

All data are presented as mean ± SEM for experiments including numbers of mice or duplicates as indicated in figure legends. Statistical analyses were conducted using the GraphPad Prism software version 6.0 (GraphPad Sofeware, CA, USA) for Windows. The two-tailed Student’s test or analysis of variance (one-way ANOVA) was used to evaluate statistical differences; p∗ < 0.05 was considered statistically significant.

## Data Availability

• RNA seq data have been deposited at SRA and are publicly available as of the date of publication. Accession numbers are listed in the key resources table. • This paper does not report original code. • Any additional information required to reanalyze the data reported in this paper is available from the lead contact upon request.
