# Supplementary material for: Deletion of RNF186 expression suppresses diet-induced hepatic steatosis by regulating insulin activity
Source: iScience. 2022 Feb 2;25(2):103859. doi: 10.1016/j.isci.2022.103859 (PMC8850801; doi:10.1016/j.isci.2022.103859)
Supplement: Document S1. Figures S1–S4 and Table S1 [file mmc1.pdf]

iScience, Volume *25*

Supplemental information

Deletion of RNF186 expression suppresses diet-induced hepatic steatosis by regulating insulin activity

Xiuqi Hu, Qifan Zhang, Manyu Guo, Qianqian Yuan, Xin Tong, Qing Zhang, Li Lin, Lei Zhang, Shujuan Lv, Xiaojun Liu, Chaobing Gao, Yongsheng Chang, and Huabing Zhang

Supplemental information

Supplementary Figures

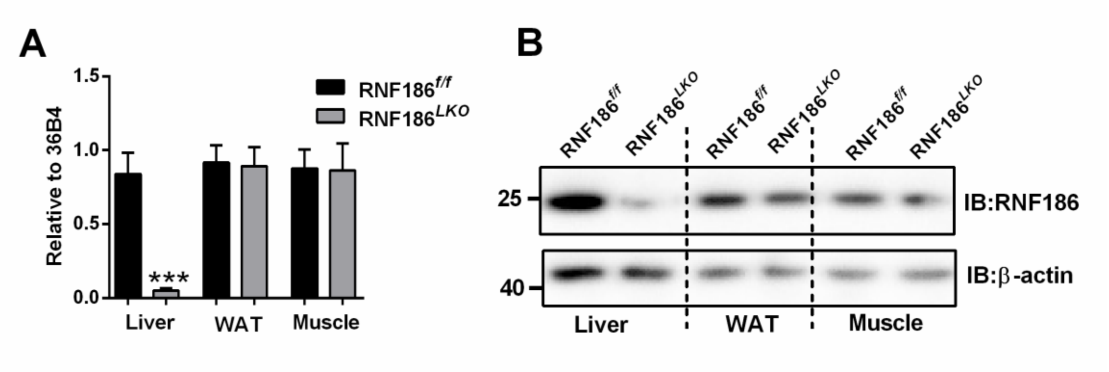

Figure S1. The expression levels of RNF186 in the different tissues of the RNF186^{LKO} and control mice. Related to Figure 1.

(A-B) qRT-PCR (A) and Western-blot (B) analysis showing mRNA and protein levels of RNF186 in the different tissues of the RNF186^{LKO} and control mice (n = 4). Data are represented as means ± SEM of three independent experiments., ***p <0.001 by Student's test.

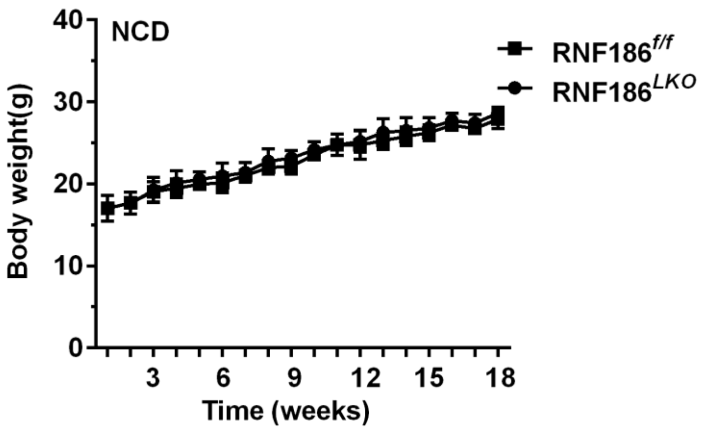

Figure S2. Body weight growth chart of WT (n=6) and liver specific knockout RNF186 (n=6) mice after treatment NCD from 1 to 18 weeks. Related to Figure 1.

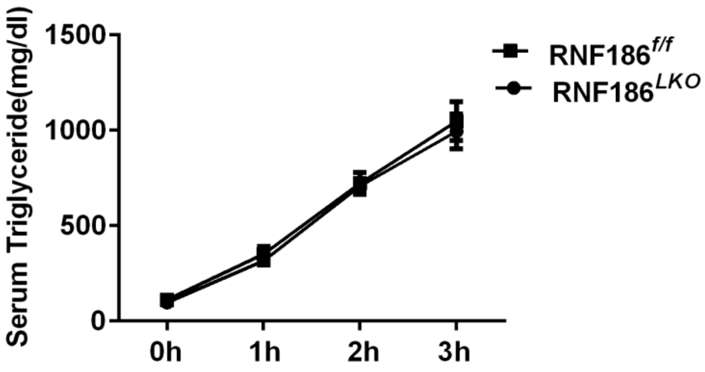

Figure S3. Deletion of RNF186 did not affect hepatic VLDL secretion in mice. Related to Figure 2.

Hepatic VLDL secretion from control and RNF186^{LKO} mice after the 18-week HFD (n = 7 per genotype). Briefly, mice fasted overnight were injected with 500 mg/kg body weight of tyloxapol via tail vein. Blood was collected by tail bleeding at several time points for serum TG measurement.

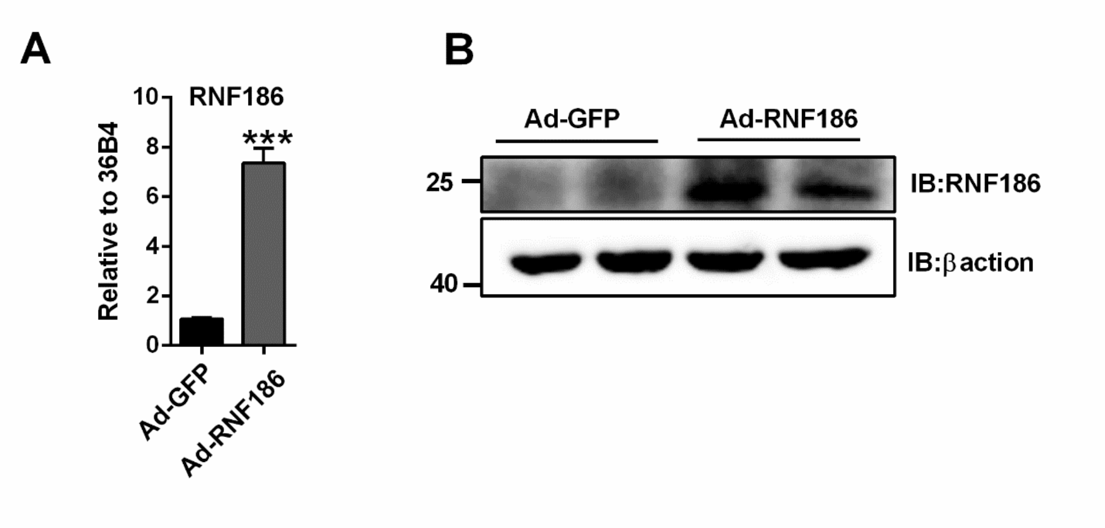

Figure S4. RNF186 adenovirus effectively increased the expression level of RNF186 in mouse liver. Related to Figure 7.

(A-B) qRT-PCR analysis showing mRNA (A) and protein levels (B) of RNF186 in the livers of Ad-GFP-or Ad-RNF186-injected C57BL/6J mice 7 days after injection (n=7/group). Data are represented as means ± SEM of three independent experiments., ***p <0.001 by Student's test.

Supplementary Tables

Table S1. RT-PCR primers. Related to STAR Methods

Mouse (gene)	Forward Primer	Reverse Primer
RNF186	5’TGTCCTCAGAGGCTAGTGG’3	5’TAGGGTGGACATCAGGAGA’3
TNFα	5’CGTCAGCCGATTTGCTATCT’3	5’CGGACTCCGCAAAGTCTAAG’3
IL-6	5’AGTTGCCTTCTTGGGACTGA’3	5’TCCACGATTTCCCAGAGAAC’3
MCP1	5’TCTGGACCCATTCCTTCTTG’3	5’AGGTCCCTGTCATGCTTCTG’3
IL-1β	5’TGAAGTTGACGGACCCCAAAA’3	5’TGATGTGCTGCTGCGAGATT’3
SREBP-1c	5’GGAGCCATGGATTGCACATT’3	5’GGCCAGGGAAGTCACTGT’3
ACC	5’AGGAAGATGGCGTCCGCTCTG’3	5’GGTGAGATGTGCTGGGTCAT’3
FAS	5’GTAAGTTCTGTGGCTCCAGAG’3	5’GCCCTCCCGTACACTCACTC’3
PPARγ	5’GCATGGTGCCTTCGCTGATGC’3	5’AGGCCTGTTGTAGAGCTGGGT’3
SCD1	5’CTGCACCTCCCTCCGGAAAT’3	5’TCCTCCAGACGTACTCCAGC’3
PGC-1α	5’TGGACGGAAGCAATTTTTCA’3	5’TTACCTGCGCAAGCTTCTCT’3
G6pase	5’AAGTCCTCTTCCGACATCCAG’3	5’GTCTCACAGGTGACAGGGAAC’3
PEPCK	5’CAGGATCGAAAGCAAGACAGT’3	5’AAGTCCTCTTCCGACATCCAG’3
PPARα	5’ACAAGGCCTCAGGGTACCA’3	5’GCCGAAAGAAGCCCTTACAG’3
CPT-1a	5’GAACCCCAACATCCCCAAAC’3	5’TCCTGGCATTCTCCTGGAAT’3
CD36	5’TCCCTTGGCAACCAACCAC’3	5’TCCACCAGTTGCTCCACAC’3
36B4	5’ATCCCTGACGCACCGCCGTGA’3	5’TGCATCTGCTTGGAGCCCACGTT’3
